# Supplementary material for: Content-rich biological network constructed by mining PubMed abstracts
Source: BMC Bioinformatics. 2004 Oct 8;5:147. doi: 10.1186/1471-2105-5-147 (PMC528731; doi:10.1186/1471-2105-5-147)
Supplement: Additional File 5 — The original Chilibot query results of the term "long-term potentiation (LTP)" and 22 other terms, limiting the latest references analyzed to the years 1990, 1995, 2000, and 2004. [file 1471-2105-5-147-S5.bz2 › chilibotAdditionalFile5/ltp2004/html/PI-3K_ACTIN.html]

 


 **PI-3K** and **ACTIN** 
  
Found 226 abstracts in PubMed,  **30 abstracts were retrieved and analyzed**.  


---

 Search Google  |
 PDF files only 
|  EDU domain only 

---

**Interactive relationship** (e.g. stimulation, inhibition, etc)

- Pretreatment of MCF7 cells with the specific PI 3 kinase  [ **PI-3K** ]  inhibitor wortmannin abolished both the activation of Rac1 and  **actin**  reorganization, while the opioid induced phosphorylation of FAK and vinculin remained unaffected.  Ref: 12878162 Exp Cell Res, 2003
- PI 3 kinase  [ **PI-3K** ]  inhibition blocks activation of Rac... and diminishes the association of Arp2 3 complex and CapZ with polymerized  **actin** .  Ref: 12788695 Am J Physiol Cell Physiol< MedlineTA>Am J Physiol Cell Physiol, 2003
- Our findings place PI 3 kinase  [ **PI-3K** ]  activity upstream of Rac, gelsolin and Arp2 3 complex activation induced by FcgammaRIIA and clearly distinguish the FcgammaRIIA signaling pathway to  **actin**  filament assembly from the thrombin receptor PAR 1 pathway.  Ref: 12788695 Am J Physiol Cell Physiol< MedlineTA>Am J Physiol Cell Physiol, 2003
- The localized  **actin**  polymerization is independent of PI3 kinase  [ **PI-3K** ]  and rho protein activity and requires Arp2 3 complex and cofilin function.  Ref: 12952932 J Cell Biol, 2003
- Shape change,  **actin**  filament barbed end exposure and quantifiable  **actin**  assembly require phosphoinositide PI 3 kinase  [ **PI-3K** ]  activity and a rise in intracellular calcium.  Ref: 12788695 Am J Physiol Cell Physiol< MedlineTA>Am J Physiol Cell Physiol, 2003
- Furthermore, addition of constitutively active D3 phosphorylated ppIs or recombinant PI 3 kinase  [ **PI-3K** ]  subunits to octylglucoside permeablized platelets elicits  **actin**  filament barbed end exposure by releasing gelsolin and CapZ from the cytoskeleton.  Ref: 12788695 Am J Physiol Cell Physiol< MedlineTA>Am J Physiol Cell Physiol, 2003
- Lamellipodium protrusion, associated with  **actin**  polymerization at the leading edge sustained the extension of cadherin contacts through a phosphoinositide 3 kinase PI 3 kinase  [ **PI-3K** ]  Rac1 pathway.  Ref: 14657280 J Cell Sci, 2004

**Parallel relationship** (e.g. studied together, co-existance, homology, etc.)

- Cadherin adhesions were formed by PI 3 kinase  [ **PI-3K** ]  independent, Rac1 dependent co recruitment of adhesion complexes and  **actin**  filaments.  Ref: 14657280 J Cell Sci, 2004
- FGF 2 induced reorganization and disruption of  **actin**  cytoskeleton through PI 3 kinase  [ **PI-3K** ] , Rho, and Cdc42 in corneal endothelial cells.  Ref: 14685150 Mol Vis, 2003
- We investigated whether FGF 2 plays a role in this morphogenetic pathway by reorganizing  **actin**  cytoskeleton through the actions of phosphatidylinositol PI 3 kinase  [ **PI-3K** ]  and the Ras related Rho family of small guanosine triphosphatases GTPases.  Ref: 14685150 Mol Vis, 2003
- Our results collectively indicate that the  **actin**  cytoskeleton mediates NO induced regulatory effects in chondrocytes by modulating down regulation of PI 3 kinase  [ **PI-3K** ]  and Akt, activation of p38 kinase, and inhibition of PKCalpha and zeta  Ref: 12907684 J Biol Chem, 2003
- This effect is mediated by PI 3 kinase  [ **PI-3K** ]  and required integrity of  **actin**  cytoskeleton.  Ref: 12911544 Kidney Int, 2003
- In this study, the role of phosphatidylinositol PI 3 kinase  [ **PI-3K** ] , Akt protein kinase B Akt PKB, and p70 S6 kinase activation in LIF induced hypertrophic responses such as stimulation of protein synthesis, atrial natriuretic peptide ANP gene expression, and reorganization of  **actin**  filaments into sarcomeric units was investigated in cultured cardiac myocytes.  Ref: 12796566 Kobe J Med Sci, 2003
- Insulin stimulates glucose uptake by recruiting glucose transporter 4 GLUT4 from an intracellular pool to the cell surface through a mechanism that is dependent on phosphatidylinositol PI 3 kinase  [ **PI-3K** ]  PI3 K and cortical  **actin**  remodeling.  Ref: 12808101 Mol Cell Biol, 2003
- Subcellular localization of proteins was determined by immunofluorescent staining and expression of Cdk4, p27, PI 3 kinase  [ **PI-3K** ] , Akt, and beta  **actin**  was analyzed by immunoblot analysis.  Ref: 12939297 Invest Ophthalmol Vis Sci, 2003
- However, in many cell types, there is a correlation between  **actin**  polymerization, activation of phosphatidylinositol 3 kinase PI 3 kinase  [ **PI-3K** ] , and the production of the second messenger phosphatidylinositol, 5 triphosphate.  Ref: 12834623 Exp Mol Pathol, 2003
- Interestingly, pretreatment with the PI 3 kinase  [ **PI-3K** ]  inhibitor wortmannin 100 nM prior to retinoic acid treatment prevented retinoic acid induced  **actin**  reorganization and cell enlargement.  Ref: 12834623 Exp Mol Pathol, 2003
